# The Influence of Transformational Teacher Leadership on Academic Motivation and Resilience, Burnout and Academic Performance

**DOI:** 10.3390/ijerph17207687

**Published:** 2020-10-21

**Authors:** Rubén Trigueros, Ana Padilla, José M. Aguilar-Parra, Isabel Mercader, Remedios López-Liria, Patricia Rocamora

**Affiliations:** 1 Department of Psychology, Health Research Centre, Hum-878 Research Team, University of Almería, 04120 Almería, Spain; rtr088@ual.es (R.T.); jmaguilar@ual.es (J.M.A.-P.); 2 Research Center Hablame, 04005 Almeria, Spain; anapadilla@centrohablame.com; 3 Department of Nursing, Health Research Centre, Physiotherapy and Medicine, University of Almería, 04120 Almería, Spain; rocamora@ual.es

**Keywords:** teacher leadership, motivation, resilience, burnout, academic performance

## Abstract

Currently, the university failure rate is around 33% of students starting their studies. Among the main reasons are demanding academic situations and the use of inappropriate coping strategies. Therefore, the aim of this study was to analyze the influence of teacher leadership on academic resilience and motivation, burnout, and academic performance. This study involved 3354 university students. A structural equation model was made to analyze the predictive relationships between the study’s variables. The results showed that teacher leadership positively predicted academic resilience and motivation; academic resilience negatively predicted burnout and positively predicted academic performance; likewise, academic motivation negatively predicted burnout and positively predicted academic performance; finally, burnout negatively predicted academic resilience.

## 1. Introduction

University academic failure is one of the issues that should be of most concern to the educational community since education is a basic instrument for the social and economic evolution of a country [1]. In this sense, 33% of Spanish students do not finish the university course in which they enrolled, 21% leave university without obtaining a degree, and the remaining 12% change studies. These data place Spain among the first countries in the European Union with the highest rate of university dropouts (11% on average) and the highest rate of change of studies (9% on average). This situation also represents a loss of EUR 1500 million from the Spanish public coffers, which could be used to promote public education [2]. This premature dropout from university studies can have serious consequences for both the individual and society. In this sense, an early dropout will lead to major educational deficiencies, making it difficult to find a job in the future. In addition, they will show a greater propensity to work illegally and a greater demand for social services. On the other hand, the social consequences of dropping out of university are: (a) problems of social integration, (b) income inequality, and (c) low tax rates for the state. Among the main reasons for this abandonment, students cite lack of capacity and time, low motivation, high anxiety, absenteeism, and loss of interest [3]. Thus, students’ classroom experiences can be key to staying in university and completing their studies [4]. In this sense, teachers have considerable potential to influence the motivational, affective, and behavioral responses of their students, exercising the role of leader within the classroom [5]. Therefore, this study aimed to analyze the influence of teacher leadership on motivation, academic resilience, burnout, and student academic performance.

### 1.1. Teacher Leadership

One of the main theories that have tried to explain the effect of leaders’ behaviors on followers is Transformational Leadership Theory [6]. This theory states that transformational leaders have a positive influence on followers because they are able to inspire them to move into action on the basis of a shared vision, aligning vision and objectives with the needs and goals of the group of followers [7]. In this sense, transformational leaders seek to discern the needs, strengths, and goals of the followers in order to be in tune with them [8]. In addition, transformational leaders seek to stimulate followers to think for themselves and in new ways in order to overcome challenges [7].

The theory of transformational leadership has been present in many studies and different contexts, some examples are: in the workplace [9], organizational contexts [10], crime [11], and also in the educational field [8]. It is in the latter context that transformational leadership theory has most recently arrived, giving rise to what it calls “Transformative Teaching” [8]. This style of teaching suggests that the transformational behaviors displayed by teachers enhance student engagement in the classroom. In this sense, those teacher behaviors that deviate from self-interest, and instead seek to inspire students, will lead to higher levels of functioning, increasing their interest and participation in the classroom, and encouraging their critical thinking.

Current studies in the educational context showed that teacher leadership in the classroom was positively related to critical thinking [12], academic performance [13], motivation [14], and classroom engagement [13]. However, there are currently no studies that have analyzed how classroom leadership favors university students’ ability to adapt to academic difficulties since current studies have focused more on aspects outside the educational context.

### 1.2. Academic Motivation

Motivation refers to the different reasons that drive certain actions and guide human behavior. In this sense, motivation includes both energy and direction, persistence and purpose of behavior, and includes actions and intentions [15]. However, motivation is not something that occurs spontaneously, but rather the experiences of the individual and the social context play an important role [16]. The Self-Determination Theory states that motivation can be of three different types: demotivation, extrinsic motivation (includes four types of regulation), and intrinsic motivation [17].

Amotivation refers to the lack of interest in the activity, lacking motivation. External motivation refers to the fact that what drives the individual comes from the outside. Within external motivation, external regulation refers to the individual’s commitment to avoid a punishment or get a reward; introjected regulation refers to the individual’s search for positive feelings towards him or herself or the avoidance of negative emotions; identified regulation refers to the individual’s commitment to the activity because he or she feels identified with it; integrated regulation refers to when the individual’s behavior and conduct are prioritized according to internal values and needs; finally, there is intrinsic motivation which refers to the commitment to the activity simply for the sake of enjoyment and fun.

Studies that have analyzed the influence of internal motivation in academia showed that it is positively related to adaptive behaviors [18], academic performance [19], metacognitive processes [20], and critical thinking [20]. Conversely, external motivation and amotivation showed to be positively related to school dropout [21], stress [22], and exam failure [23].

### 1.3. Academic Resilience

Resilience is understood as a psychological element or capacity that people possess in order to overcome extreme situations and the negative effects to which they are exposed [24]. In a way, resilience represents one of the main psychological characteristics that are related to psychological and emotional well-being and academic success. 

Throughout their student period, students are exposed to academic experiences and situations (both negative and positive) to which they have to adapt, reacting in one way or another to an adverse situation, and may or may not overcome it [25]. Those students who succeed show a resilient attitude that allows them to adapt and overcome these negative experiences [26]. In this way, resilience is understood as a construct that is linked to protective and vulnerable factors within and outside the individual, which influence the individual’s adaptation to vicissitudes and stressful events that result in a loss of homeostasis [27]. However, resilience is not a construct that is inherited by the subject, nor does it remain unchanged, but is acquired and evolves from different life situations and the control of emotions [28].

Until now, studies of resilience in academic contexts have been positively related to intrinsic motivation [29], academic goals [30], academic performance [30], and internal well-being [31]. In contrast, resilience has been negatively associated with stress [25], anxiety [25], and school failure [32]. However, studies on resilience in the university context are very scarce, as most studies in academic contexts focus on the secondary school stage, despite the fact that the university context exceeds the secondary school stage in terms of dropouts.

### 1.4. Burnout

During the training process, many students develop a range of skills that help them to achieve their academic goals, but other young people have difficulty in adapting to the difficulties that prevent them from achieving their goals [33]. Thus, some students succeed in developing appropriate strategies to cope with academic demands. In contrast, others do not and become unable to modify the problematic situation, resulting in the use of escape or avoidance behaviors as forms of coping that are not necessarily appropriate in this situation [34].

In the face of the continuous presence of demanding academic situations and the use of coping strategies such as escape or avoidance, it can contribute to generating sensations of not being able to give more of oneself, both physically and psychologically, a negative attitude of criticism, devaluation, loss of interest in transcendence, of value in the face of study, and growing doubts about one’s capacity to carry it out [35]. The simultaneous presence of these manifestations is known as academic burnout syndrome.

Studies to date in the academic context, which have focused on burnout, showed that it is a good predictor of poor academic performance [36], frustration with studies [37], and reduced student self-concept [38]. These results showed that burnout played an important role as a predictor of various variables related to the academic life of university students. However, it is necessary to point out that the association between academic burnout and academic performance is still not clear since some understand low performance as a predictor variable of the syndrome, whereas others understand it as a consequence of it [39]. Therefore, this study aimed to incorporate protective factors such as resilience and internal motivation as elements that can clarify whether burnout predicts academic performance.

### 1.5. Objectives and Hypothesis

According to the above, the aim of this study was to analyze the influence of teacher leadership on academic resilience and motivation, burnout, and academic performance. To this end, the following hypotheses are specified: (a) teacher leadership will positively predict academic resilience and motivation; (b) academic resilience will negatively predict burnout and positively predict academic performance; (c) academic motivation will negatively predict burnout and positively predict academic performance; (d) burnout negatively predicts academic resilience.

## 2. Method

### 2.1. Participants

The sample of participants in the study was 3354 university students, consisting of 1653 men and 1701 women (Table 1). The students were enrolled at the universities of Granada and Almeria. Their ages were between 18 and 31 years (M = 22.36; SD = 1.88). The sampling technique was non-probabilistic and incidental based on those university centers we had access to and the students who wanted to participate. To be able to participate in the study, it was necessary to give the informed consent, signed by the parent or legal guardian, and to participate voluntarily.

### 2.2. Measurements

Leadership in the classroom: The Spanish version [40] of the Transformative Teaching Questionnaire [41] was used. This questionnaire consisted of 16 items distributed equally among 4 factors (individualized consideration, idealized influence, intellectual stimulation, and inspirational motivation). The participants in the study completed the questionnaire using a Likert scale from 1 (nothing at all) to 5 (frequently). This scale has been used in numerous studies to evaluate teacher leadership in the classroom (e.g., [42,43]).

Academic Resilience: The Spanish version [44] of the Academic Resilience Scale [45] was used. There were 30 items on the scale, which were distributed among three factors (reflection and adaptation of the search for help, perseverance, negative affect, and emotional response). Those items that had a positive connotation were reversed. Thus, a high score indicated greater academic resilience. The participants in the study completed the questionnaire by means of a Likert scale from 1 (probable) to 5 (improbable).

Academic motivation towards learning: The [46] version was used. The scale had 30 items distributed among six factors: demotivation, extrinsic motivation (external regulation, introjected regulation, identified regulation, and integrated regulation), and intrinsic motivation. The participants in the study completed the questionnaire using a Likert scale from 1 (not true) to 7 (completely true). In the present study, it was necessary to calculate the Self-Determination Index (SDI) in order to know the level of academic motivation of the students. This index has been used in several similar studies (see [47]).

Burnout: The Spanish version [48] of the Maslach Burnout Inventory [49] was used. There are two items that make up the scale distributed among three factors: personal fulfilment, depersonalization, and emotional exhaustion. The participants in the study completed the questionnaire using a Likert scale from 0 (never) to 6 (every day).

Academic Performance: The grade at the end of the academic year was taken into consideration. The grade was coded as follows: outstanding (5), notable (4), good (3), passed (2), and failure (1).

### 2.3. Procedure

In order to carry out the study, the management and teaching staff of the universities were initially contacted. They were informed of the objective of the study, and their collaboration was requested in order to carry out the study. Subsequently, the university students that agreed to take part in the study were contacted and informed of the objective of the study and asked to participate. The questionnaires were completed individually, anonymously, and at the beginning of the classes. At the beginning of the completion of the questionnaires, it was stressed that it was not compulsory to participate in the study and that a member of the research group should be present to resolve any doubts that might arise. The recommendations of the American Psychological Association were followed for this study. In addition, ethical approval was obtained (Ref. UALBIO 2019/014) by the Research Committee of the University of Almería (Spain). 

### 2.4. Data Analysis

The SPSS v.25 statistical package (IBM, Armonk, NY, USA) was used to perform various statistical analyses (mean, standard deviation, reliability analysis, and Pearson’s bivariate correlations). Similarly, the AMOS v.20 statistical package (IBM, Armonk, NY, USA) was used to analyze the predictive relationships between the variables of the study, through a structural equation model (SEM). For the SEM, a bootstrapping of 6000 interactions and the maximum likelihood procedure were used. The estimators were considered robust despite the lack of normality, so they were not affected. The adjustment rates taken into account for SEM were as follows [50] (Table 2):

According to Marsh, Hau, and Wen [51], these adjustment rates must be taken into account with some caution as they can be very restrictive or difficult to achieve in overly complex models.

## 3. Results

### 3.1. Preliminary Analysis

As can be seen in Table 3, the bivariate correlations were positive among all factors, except when correlated to the burnout. In addition, the reliability analysis reflected a score above 0.70 [52], so they were considered robust.

### 3.2. Structural Equation Model Analysis

**Figure 1:**
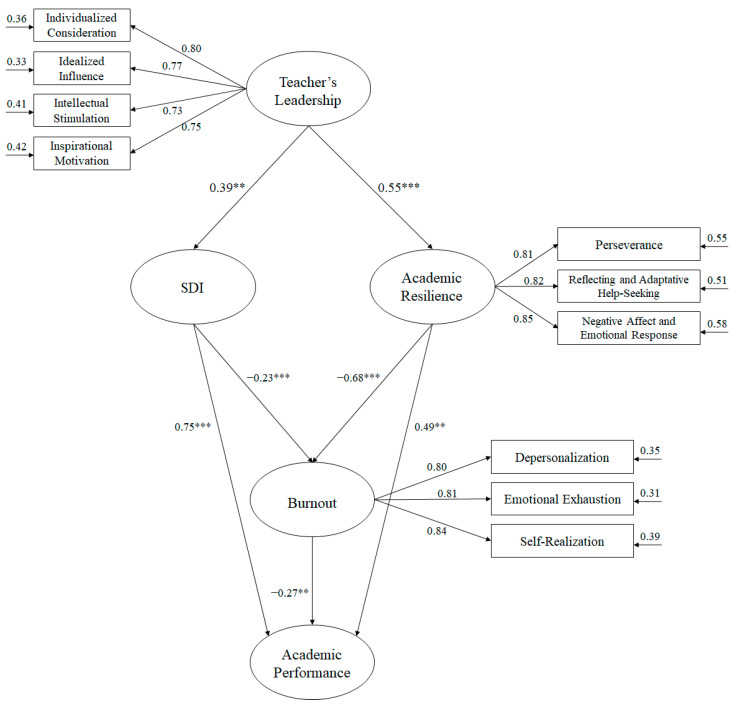
Hypothesized model, where all variables are related to one another. All parameters are standardized and are statistically significant. Note: *** *p* < 0.001; ** *p* < 0.01.

The fit rates of the structural equation model (Figure 1) that were used to analyze the predictive relationships, as described in the hypotheses, showed adequate scores: χ^2^ (49, N = 3354) = 140.03, χ^2^/df = 2.86, *p* < 0.001, IFI = 0.96, TLI = 0.96, CFI = 0.96, RMSEA = 0.055 (IC 90% = 0.047–0.058), SRMR= 0.049. These parameters showed an acceptable fit, and therefore the hypothesized model can be considered adequate. The relationship between the study variables was examined through standardized regression weights.

## 4. Discussion

This study sought to analyze the influence of teacher leadership on motivation, academic resilience, burnout, and academic performance of university students. For the first time, this study looked at the direct relationship of teacher leadership on motivation and academic resilience of university students. Both psychological elements are essential for their involvement and participation in the classroom, allowing them to develop their skills and knowledge through a balance between discovery and previous experiences [53]. Similarly, this study looked at the direct relationship between motivation and academic resilience with respect to burnout. In this sense, the change from secondary school to university, together with the academic pressure between jobs and exams, subjected students to a high level of stress that could lead to emotional exhaustion [25]. Therefore, both factors, motivation and resilience, were considered as protective elements that can help the consolidation and creation of skills and coping strategies in the face of possible disruptive events [54].

The results of this study showed that teacher leadership positively predicted motivation and academic resilience. These results can only be compared with similar studies from the secondary level, albeit in a segmented way [55]. In this regard, a study by Bolkan, Goodboy, and Griffin [56], with secondary school students showed that those teachers who were perceived as transformational leaders by their students had high levels of intrinsic motivation and were more involved in various classroom activities. Similarly, a physical education study of secondary school students showed that those students who were most interested and motivated in learning were those who perceived teachers as leaders [57]. In contrast, there is currently no evidence of studies in education that have linked teacher leadership to academic resilience in students. However, there are a few studies in the field of employment. One study by Nguyen, Kuntz, Näswall, and Malinen [58] that used a sample of workers in the financial sector showed that workers with high levels of resilience and high adaptive capacity were perceived as good leaders by their bosses. Similarly, a study that used a sample of technology workers showed that those bosses who were transformational leaders had positive relationships with a positive work climate, receptive workers, and increased work involvement [59]. These results highlighted the importance and role of teachers in the classroom. In this sense, those teachers who act as transformational leaders for their students will tend to develop a greater capacity to adapt to academic difficulties and a greater interest in the classroom. This requires that teachers pay attention to the interests of their students, show understanding, listen to them, and know their goals and objectives in order to provide them with a fulfilling educational experience. 

Likewise, the results showed how academic motivation predicted burnout negatively and academic performance positively. These results were similar to several studies in the academic field, including the university one, where motivation was a positive predictor of academic performance and a negative one of burnout (e.g., Stoeber, Childs, Hayward, and Feast, [60]). Similarly, a study conducted by Chang, Lee, Byeon, and Lee [61] with high school students showed that those students who showed high internal motivation predicted burnout negatively and perfectionism positively predicted burnout. In this sense, perfectionism is an egomaniacal attitude of the individual, as opposed to the Self-Determination Theory postulates regarding internal motivation [62]. Similarly, a study carried out with university students showed that motivation towards learning was positively related to academic performance and participation, and negatively to burnout [63]. Furthermore, the results showed how resilience predicted burnout negatively and academic performance positively. However, these results can only be compared with a few studies in the educational field and in a fragmented way. In this regard, a study by Dunn, Iglewicz, and Moutier [64] with medical students showed that students with high levels of resilience had a greater sense of psychological well-being, as well as acted as a protector against burnout. Similarly, a study with nursing students [65] showed that students with high levels of resilience experienced low levels of burnout and high levels of psychological well-being. On the other hand, there are several studies in the educational field that have linked resilience to academic performance. A study conducted by Kotzé and Kleynhans [66] with five-year-old students showed that those students who showed greater maturity were associated with greater resilience, which in turn was positively related to academic performance. Similarly, Kwek, Bui, Rynne, and So [67] in their study with university students showed that those with high self-esteem and academic performance were positively related to academic performance. These results showed that intrinsic motivation and resilience could be considered as protective psychological factors against stressful events and various problem situations faced by students. Therefore, it is essential that classes create a climate in which internal motivation, achievable goals, and surmountable challenges predominate so that students can extrapolate the positive experiences that take place during classes and bring them into the academic context.

Finally, the results showed how the burnout negatively predicted academic performance. These results were similar to several studies in the university academic field [68,69,70]. In this regard, a study conducted by Salanova, Schaufeli, Martínez, and Bresó [68] with university students found that the vicissitudes perceived by students were positively associated with burnout, whereas facilitators were negatively associated with burnout. In addition, burnout was negatively related to academic performance. Similarly, a study conducted by Yang [71] revealed that student burnout had a negative effect on academic performance. These results can be explained by the fact that those students who perceived many obstacles felt more burnout, affecting their academic future, as the problems caused by the obstacles interfered with academic performance. As a result of the findings of this study, it is obvious that there is a need to control student burnout to improve interest in learning and academic performance. This requires teachers and schools to design and plan courses, tests, and even career plans with great care to reduce student burnout and improve academic performance, and ultimately the attainment of a university degree.

Despite the results achieved in this study, it is necessary to highlight some limitations. The present study was based on the use of self-reporting questionnaires, which limits the information to be collected. Furthermore, the relationships between the variables can be interpreted in many different ways depending on the reader’s opinions. Finally, the selection of participants was probably not incidental; therefore, no comparisons can be made between study populations. On the other hand, future studies should analyze the influence of close family and friends on academic burnout, resistance, and motivation. In this sense, family and friends constitute the nucleus where the first experiences of socialization, protection, and security originate, being the origin of the future behavior of the adolescent based on previous learning [72].

## 5. Conclusions

In short, the results achieved in this study showed that teacher leadership positively predicted academic resilience and motivation. In turn, academic resilience and motivation predicted burnout negatively and academic performance positively. These results showed that academic contexts could generate both positive and negative situations in the classroom and that the role of the teacher is key to a positive transformation in students’ experiences in the classroom.

## Figures and Tables

**Table 1:** Description of the sample.

University	Degree	Distribution Sample
University of Granada	Education	207 men and 254 women
	Psychology	214 men and 252 women
	Sciences	164 men and 198 women
	Humanities	201 men and 203 women
University of Almeria	Education	222 men and 216 Women
	Psychology	259 men and 241 women
	Sciences	237 men and 175 women
	Humanities	149 men and 162 women
		N = 3354

**Table 2:** Adjustment indexes.

Statistics Indexes	Adequate Scoring
χ^2^/df	Between 2 and 3
CFI (Comparative Fit Index)	Over 0.95
IFI (Incremental Fit Index)	Over 0.95
TLI (Tucker Lewis Index)	Over 0.95
RMSEA (Root Mean Square Error of Approximation) plus your confidence interval (CI) at 90%	Equal to or less than 0.06
SRMR (Standardized Root Mean Square Residual)	Equal to or less than 0.08

**Table 3:** Descriptive statistical analysis and reliability analysis.

Factors	*M*	*SD*	α	1	2	3	4	5
1. Teacher Leadership	3.01	0.87	0.76	-	0.43 ***	0.52 ***	−0.47 ***	0.39 ***
2. Academic Resilience	3.31	0.87	0.84		-	0.68 ***	−0.53 ***	0.42 ***
3. SDI-Academic	14.05	9.51	-			-	−0.24 **	0.33 **
4. Burnout	2.67	1.21	0.83				-	−0.31 **
5. Academic Performance	3.82	1.33	-					-

*** *p* < 0.001; ** *p* < 0.01, SDI: Self-Determination Index.
